# Quadruple 9-mer-based protein binding microarray with DsRed fusion protein

**DOI:** 10.1186/1471-2199-10-91

**Published:** 2009-09-18

**Authors:** Min-Jeong Kim, Tae-Ho Lee, Yoon-Mok Pahk, Yul-Ho Kim, Hyang-Mi Park, Yang Do Choi, Baek Hie Nahm, Yeon-Ki Kim

**Affiliations:** 1 Department of Agricultural Biotechnology, Seoul National University, Seoul 151-921, Korea; 2 GreenGene Biotech Inc., Myongji University, Yongin 449-728, Korea; 3 Division of Bioscience and Bioinformatics, Myongji University, Yongin 449-728, Korea; 4 National Institute of Crop Science, Suwon 441-857, Korea

## Abstract

**Background:**

The interaction between a transcription factor and DNA motif (*cis*-acting element) is an important regulatory step in gene regulation. Comprehensive genome-wide methods have been developed to characterize protein-DNA interactions. Recently, the universal protein binding microarray (PBM) was introduced to determine if a DNA motif interacts with proteins in a genome-wide manner.

**Results:**

We facilitated the PBM technology using a DsRed fluorescent protein and a concatenated sequence of oligonucleotides. The PBM was designed in such a way that target probes were synthesized as quadruples of all possible 9-mer combinations, permitting unequivocal interpretation of the *cis*-acting elements. The complimentary DNA strands of the features were synthesized with a primer and DNA polymerase on microarray slides. Proteins were labeled via N-terminal fusion with DsRed fluorescent protein, which circumvents the need for a multi-step incubation. The PBM presented herein confirmed the well-known DNA binding sequences of Cbf1 and CBF1/DREB1B, and it was also applied to elucidate the unidentified *cis*-acting element of the OsNAC6 rice transcription factor.

**Conclusion:**

Our method demonstrated PBM can be conveniently performed by adopting: (1) quadruple 9-mers may increase protein-DNA binding interactions in the microarray, and (2) a one-step incubation shortens the wash and hybridization steps. This technology will facilitate greater understanding of genome-wide interactions between proteins and DNA.

## Background

Transcription factors (TFs) are regulatory proteins that interact with specific DNA sequences to control gene expression. The DNA binding domains of TFs bind to specific upstream sequences (*cis*-acting elements) of target genes and modulate the transcription process. Protein-DNA binding properties have been investigated by traditional procedures, such as the Electrophoretic Mobility Shift Assay (EMSA) and filter binding assay [1,2]. However, these methods are labor-intensive and are restricted to the intended application in that they are usually designed with prior knowledge obtained via promoter-reporter assays. Comprehensive genome-wide methods, along with the availability of whole-genome sequences and advances in microarray technology, have been developed to characterize protein-DNA binding specificities [3].

Some of well-known high-throughput methods are chromatin immunoprecipitation (ChIP)-chip, DNA adenine methyltransferase identification (DamID), protein microarray and protein binding microarray (PBM) [4-13]. ChIP-chip is a combinational procedure of chromatin immunoprecipitation and DNA microarray experiment. To enrich protein-bound DNA fragments, cells are treated with a reagent to form cross-links between DNA and a protein, typically formaldehyde, and immunoprecipitated with a protein-specific antibody. The enriched DNA fragments are labeled with a fluorescent dye by PCR amplification and hybridized to DNA microarrays. Many ChIP-chip studies have been performed for transcription factors [4], RNA polymerases [5,6] and replication-related proteins [7] to identify their recognition sequences. ChIP-chip might be applicable under the restrictions of the antibodies available for each protein.

DamID has been applied to survey *in vivo *binding sites of a protein with combination of targeted DNA methylation and microarray. Dam is a DNA methyltransferase, which can be targeted to specific sequences by fusion to a DNA binding protein of interest. The binding of the fusion protein leads to DNA methylation of adjacent sequence and these methylated regions can be discriminated by methyl-specific restriction enzyme. The digestion fragments are labeled with fluorescent dye by random priming and applied to microarrays [8]. Although DamID is independent of antibody, it may not be suitable to a protein which depends on post-translational modification in order to potentially interact with DNA [9].

Alternatively, protein microarray was used to identify corresponding sequences representing binding affinity against potential DNA-binding proteins which were attached on a slide [10]. However, the cloning of a vast number of proteins is a major limitation in the fabrication of protein microarrays.

PBM was introduced to conveniently determine protein-DNA interactions *in vitro *[11]. The whole-genome yeast intergenic microarray is prepared by spotting double-stranded DNA. Separately, glutathione S-transferase (GST)-tagged proteins of interest are expressed, purified and applied to microarrays. The protein-bound microarrays are labeled with Alexa 488-conjugated antibody to GST and fluorescent images are obtained with microarray scanner. The DNA binding sequence specificities of three transcription factors are identified by these PBMs.

More recently, PBM was improved by adapting de Bruijn sequences and in situ synthesis of DNA oligonucleotides on slide [12]. The de Bruijn sequences represent not only all contiguous 10-mers, but also all 10-mers with a gap size of 1 nucleotide. The double-stranded microarrays were prepared by primer extension and GST-tagged proteins applied to the slides. The protein-bound microarray was stained with a fluorophore-conjugated polyclonal antibody against GST, and binding strength was analyzed based on the fluorescence intensity to determine the consensus sequence. This technology has proven to be useful with well-known TFs, such as Cbf1 (centromere binding factor 1 from yeast), Zif268 (C2H2 zinc fingers from mouse), and Oct-1 (POH homeodomain from human). The researchers showed known 8-mer or extended motifs by computing rank-based statistics between the *k*-mer-containing and non-containing groups. They successfully overcame the variability associated with the compact design, which might confound direct assignment of preferences between *k*-mers. Additionally, a recent study characterized the protein-DNA binding specificities of Apicomplexan AP2 (ApiAP2) putative transcriptional regulators in malaria-causing parasites using PBM technology [13].

Here we demonstrate PBM can be conveniently performed using a DsRed-monomer fluorescent protein and quadrupled oligomer sequences. The wild-type DsRed was cloned from *Discosoma sp*. reef coral and displays a tendency to aggregate tetrameric structure [14,15]. However, the folding structure of DsRed is identical to that of avGFP consisting of 11-stranded β-barrel. Also, as a mutated variant, DsRed-monomer has been used to examine subcellular localization of the tagged proteins because DsRed-monomer is monomeric and stable.

## Results

### Design of the Q9-protein binding microarray (PBM)

**Figure 1:**
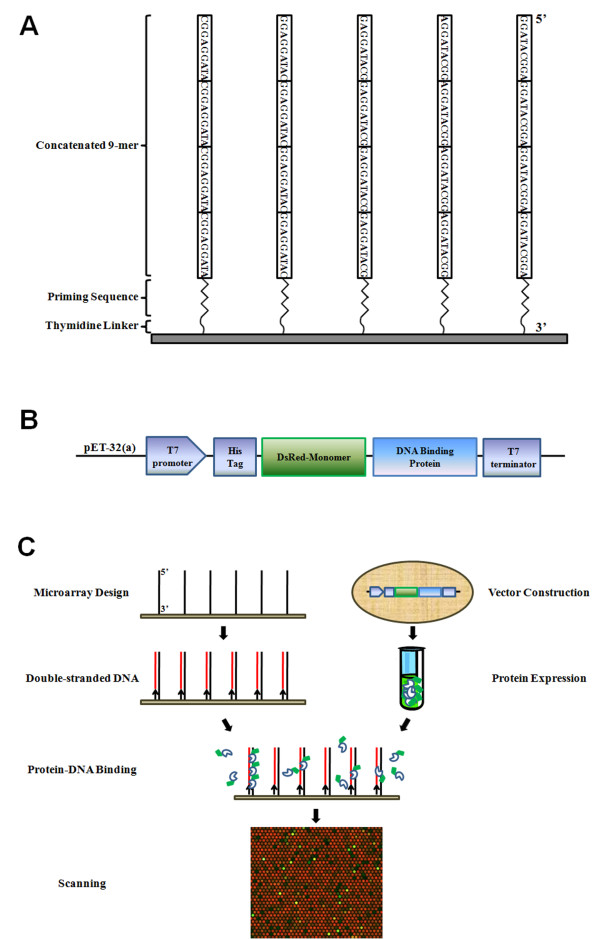
**Experiment using the Quadrupled 9-mer Protein Binding Microarray (Q9-PBM)**. (A) All possible combinations of 9-mer oligonucleotides were quadrupled, and then followed by a primer binding sequence and a 5-nt thymidine linker attached to the slide. A total of 232,145 probe features, including 131,072 features from all possible 9-mers and 101,073 replicated features, were designed. (B) The pET32-DsRed expression vector was constructed. Proteins were expressed with an N-terminal fusion to the polyhistidine-tag and red fluorescent protein (DsRed). The DsRed fluorescent protein was cloned from *Discosoma *sp. based on homology with the green fluorescent protein (GFP). DsRed possesses a similar spectrum to Cy3 that is compatible with the microarray scanner. The full length cDNA of the transcription factors was cloned into the pET32(a) expression vector followed by DsRed fluorescent protein. (C) The reverse complimentary strand was synthesized on a slide by thermo-stable DNA polymerase. A small quantity (1.6 μM) of Cy5 fluorescent dUTP was incorporated to confirm successful elongation. Purified DsRed fusion protein was incubated with the double-stranded microarray. The consensus sequence was determined from the fluorescence intensity of the spot without any further step like antibody labeling. The presented scanning image is the part of the Cbf1 result.

We designed a PBM, which we refer to as Q9-PBM, in such a way that target probes are synthesized as quadruples of all possible 9-mer combinations. A total of 131,072 features were selected after consideration of the reverse complimentary sequences of all 9-mer combinations, and 101,073 features were replicated to confirm the binding consistency. Each 9-mer was quadrupled and linked to a PCR-primer binding site following five thymidine linkers to the slide (Figure 1A). These repetitive sequences provide highly consistent results by which consensus binding motifs can be extracted, thereby allowing unequivocal interpretation. The microarray was manufactured by Agilent technology, and the reverse complementary DNA strand of each probe was synthesized on the slide via thermo-stable DNA polymerase.

### Expression of DsRed-fused transcription factors and determination of binding motifs

In the present report, all TFs were expressed with an N-terminal fusion to DsRed fluorescent protein (Figure 1B). Full-length *Cbf1 *(Centromere Binding Factor 1) and *CBF1*/*DREB1B *(C-repeat-binding factor1/dehydration-responsive element binding factor 1B) were amplified from the *S. cerevisiae and A. thaliana *genomes, respectively, and full-length *OsNAC6 *(NAM, ATAF, and CUC) was amplified from *Oryza sativa *cDNA by PCR [16-18]. All amplified clones were inserted in the pET32-DsRed recombinant vector, sequenced to verify the absence of mutations in the DNA-binding domains, and introduced into *Escherichia coli *strain BL21-CodonPlus for protein expression.

**Figure 2:**
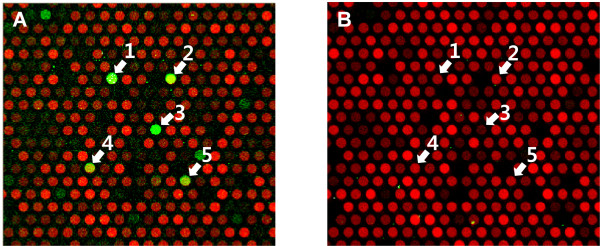
**Scan images of protein binding microarray**. DsRed-fused Cbf1 PBM image was compared to that of DsRed-monomer. DsRed was displayed in green color in contrast with red probes. Cy5 intensity depends on the base composition of probes because Cy5 fluorescent dUTP was incorporated during the synthesis of complimentary DNA strands. The arrows indicate corresponding quadrupled 9-mer sequences of probes in each microarray. 1, GTCACGTGA; 2, ACACGTGTG; 3, CGTGGGCCA; 4, ACACCCGTG; 5, GATCACGGG. (A) DsRed-fused Cbf1 was efficiently bound to the double-stranded probes. Because 'CACGTG' sequence is a Cbf1 binding motif [16], any quadrupled 9-mer probe including 'CACGTG' showed higher DsRed fluorescent intensity than other variant probes. (B) There was no specific binding of DsRed without fusion of DNA binding protein.

The complementary DNA strand was synthesized by primer extension according to the previous report [12]. The resulting microarray was scanned and Cy5 red spots throughout the microarray suggested reverse complementary strands are successfully synthesized. DsRed-fused DNA binding protein was applied to the double-stranded Q9-PBM, and the fluorescence intensity of the bound protein was acquired using a microarray scanner (Figure 1C). The Cbf1 PBM image shows that DsRed-fused Cbf1 was efficiently targeted to the specific double-stranded sequences (Figure 2).

**Figure 3:**
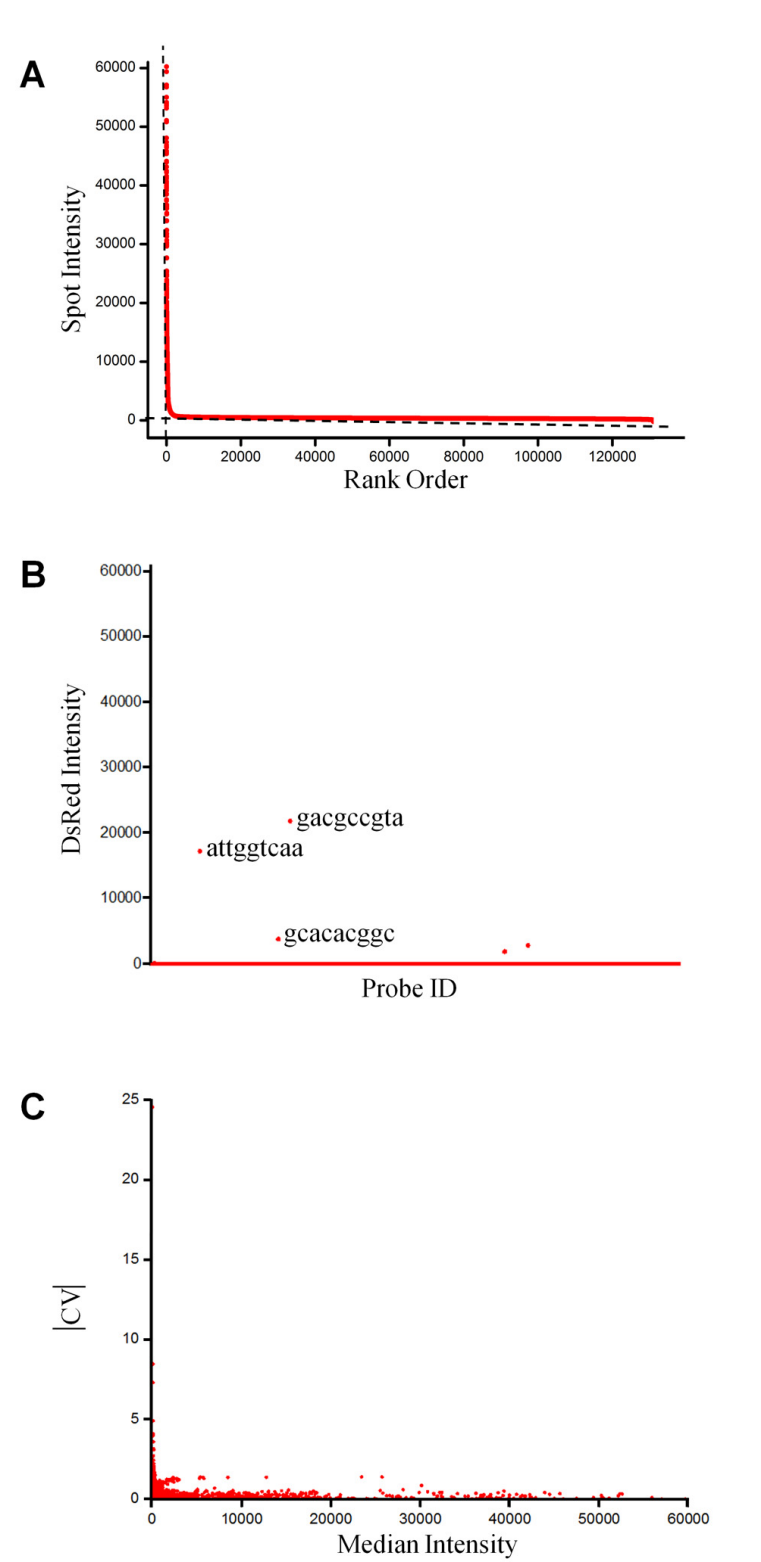
**Cy3 fluorescent signal distribution of the spot intensity**. (A) The rank-ordered signal distribution of the Cbf1 PBM shows a deep leftward slope followed by a heavy right tail because the signal distribution is due to a specific interaction between the protein and features on the microarray. Two independent linear models, y = ax+b, were applied in the deep and the heavy right tail regions. (B) Cy3 intensity was inspected to verify that DsRed was not binding to DNA sequences on the PBM. With the exception of non-specific binding spots showing a higher intensity signal, most spots exhibited background level intensity. (C) The coefficient of variation (CV) of 101,073 replicated probe pairs was observed to verify the binding consistency of Cbf1. Because higher intensity probes indicate more consistent binding properties, the consensus binding motif determined from the hierarchical rank order can be meaningful.

The consensus binding sequence was determined based on signal strength. In general, the rank-ordered signal distribution of the bound protein showed a deep leftward slope followed by a heavy right tail (Figure 3A), as observed in a previous report [12]. Because the probes in the deep slope region differed by only one base, we assumed that the signal distribution was due to a specific interaction between the protein and features on the microarray. Two independent linear models, y = ax+b, were applied to the deep and the heavy right tail region using R statistical language. The spot intensities were rank-ordered, and enrichment scores of 5-, 6-, and 7-mers were determined. Spots that exhibited strong intensity and high enrichment were subject to alignment. These groups were denoted with SEQLOGO [19].

### Binding evaluation of well-known transcription factors

**Figure 4:**
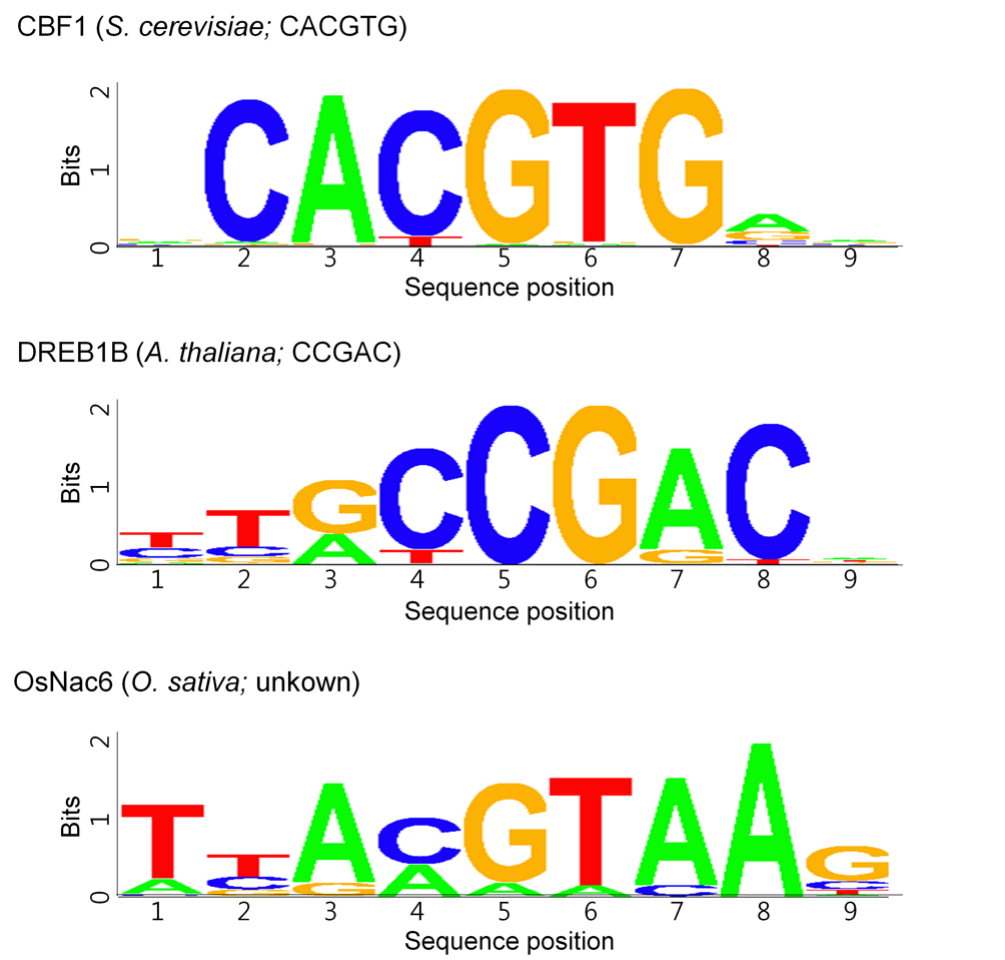
**The determined consensus binding sequences according to the PBM results**. Consensus sequences that bound robustly to each transcription factor. To determine binding motifs, two independent linear models were applied in the deep and the heavy right tail region using the R statistical language as described in Method. Organisms and previously identified consensus sequences are denoted in parentheses.

As an initial test, we verified that the DsRed protein alone did not demonstrate any significant binding to the double-stranded microarray (Figure 3B). We then inspected the results for Cbf1, a well-characterized, basic helix-loop-helix-leucine zipper family transcription factor that binds to the 'CACGTG' motif as a homodimer in yeast [16]. We observed the coefficient of variation (CV) for replicated probe pairs to verify the binding consistency in the Cbf1 microarray (Figure 3C). The CV value for high-intensity probes approached '0', which indicates that highly ranked probes reliably determine consensus-binding sequences. Based on the rank-ordered signal distribution and statistic algorithm previously described, the Cbf1 binding motif generated was 'CACGTG' (Figure 4).

We chose the CBF1/DREB1B transcription factor as another well-known example that binds to the CRT/DRE (C-repeat/cold- and dehydration-responsive DNA regulatory element) sequence in Arabidopsis [17]. CRT/DRE contains the conserved 'CCGAC' sequence, which is an important element in the promoter regions of cold-inducible genes. The CBF1/DREB1B binding sequence determined included the previously defined motif (Figure 4).

### Determination of an unknown OsNAC6 motif

**Figure 5:**
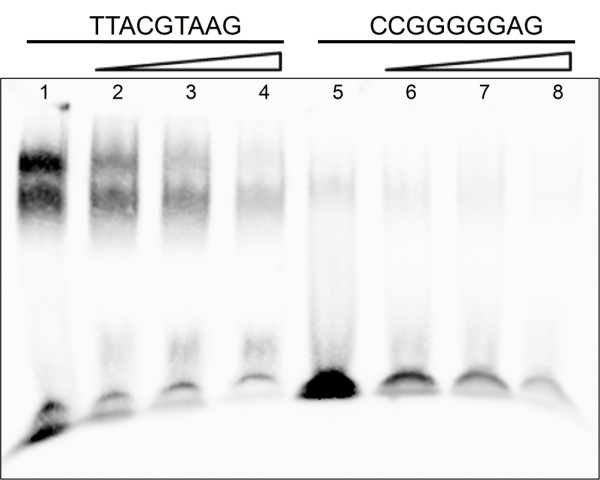
**EMSA-based competition analysis of OsNAC6**. Candidate duplexes containing 'A(A/C)GTAA' and G-rich sequences were used as core binding sequences for the interaction. The mobility shifts were performed using 5 μg of enriched OsNAC6 protein and 40 fmol of biotinylated oligonucleotides. The shift was assessed by competition using unlabeled oligonucleotides. The molar excess of competitor used was over 1,000 fold. Lane 1; OsNAC6 + biotin-labeled DNA. Lane 2; OsNAC6 + biotin-labeled DNA + unlabeled DNA (40 pmol). Lane 3; OsNAC6 + biotin-labeled DNA + unlabeled DNA (80 pmol). Lane 4; OsNAC6 + biotin-labeled DNA + unlabeled DNA (120 pmol). Lane 5; OsNAC6 + biotin-labeled DNA. Lane 6; OsNAC6 + biotin-labeled DNA + unlabeled DNA (40 pmol). Lane 7; OsNAC6 + biotin-labeled DNA + unlabeled DNA (60 pmol). Lane 8; OsNAC6 + biotin-labeled DNA + unlabeled DNA (120 pmol).

Because Q9-PBM confirmed well-known *cis*-acting elements of Cbf1 and CBF1/DREB1B, we applied the microarray to elucidate unknown binding motifs of the OsNAC6 transcription factor considered to play critical roles in abiotic and biotic stress-involved responses in *Oryza sativa*. Although the binding affinity was weaker than that observed in the former cases, we were able to determine that OsNAC6 binds not only to 'A(A/C)GTAA' (Figure 4), but also to G-rich sequences. To validate the PBM results, we chose the 9 bp candidate sequences 'TTACGTAAG' (which contains 'A(A/C)GTAA') and 'CCGGGGGAG' (which is G-rich) from the microarray and analyzed them using a gel retardation assay [20] (Figure 5). The results showed that OsNAC6 can bind to either sequence, but OsNAC6 seems to displace more of 'TTACGTAAG' motif over 'CCGGGGGAG' in our experimental setup. Additionally, we found that the presence of the 'A(A/C)GTAA' motif in the 2 kb promoter region of four rice genes (AK058583, AK105331, AK109480 and AK110725) which were previously proposed to associate with direct regulation by OsNAC6 [18].

## Discussion

Transcription factors (TFs) are regulatory proteins that interact with specific DNA sequences to control gene expression. The DNA binding domain of TFs combines with specific upstream sequences (*cis*-acting elements) of target genes and modulates the transcription rate of genes. The binding of TFs plays an important regulatory role in various metabolic pathways, developmental differentiation, and environmental responses, as well as in basal biological processes. Therefore, many applications have been developed to elucidate the interactions between TF and DNA motifs.

A genome-wide survey was conducted by Berger *et al. *using a compact microarray design [12]. They identified 8-mer or extended motifs by computing rank-based statistics between the *k*-mer-containing and non-containing groups. They successfully overcame the variability inherent to this compact design, which could have confounded the direct assignment of preferences between *k*-mers. The recently developed Agilent technology provides researchers with denser microarrays (240,000 features were included in our microarray), and we designed a 9-mer-based microarray that permits straightforward interpretation of binding sequences. We also demonstrated that DsRed-fused recombinant TFs can bind to their corresponding *cis*-acting elements. Our method provides convenient identification of protein-DNA binding interactions after a simple, one-step incubation with the microarray.

We designed a PBM, denoted as Q9-PBM, in such a way that target probes are quadruples of all possible 9-mer combinations. 131,072 features were selected from the total of 262,144 reads after consideration of the reverse complimentary sequences because a double-stranded DNA has a bidirectional aspect. The quadruple sequences can provide highly consistent and concrete results for consensus binding motifs. Our Q9-PBM employs DsRed fluorescent protein, which eliminates multiple wash and hybridization steps.

The reverse complementary DNA strand of each probe was synthesized on the slide, and DsRed-fused protein was applied to the double-stranded Q9-PBM. The rank-ordered signal distribution showed a deep leftward slope followed by a heavy right tail, suggesting a specific interaction between the protein and features on the microarray. We verified the well-known *cis*-acting elements of Cbf1 and CBF1/DREB1B, which originate from *S. cerevisiae *and *A. thaliana*, respectively. Although a direct comparison is not applicable, the Cbf1 binding intensity of Q9-PBM was compared to de Bruijn sequence-based microarray to verify the consistent binding of DsRed-fused protein [12]. In the result of de Bruijn sequence-based microarray by Berger *et al.*, totally 71 features include "RTCACGTG" sequence in their double-stranded microarray, which was referred to Cbf1 binding motif. The normalized signal intensities of these 71 features were between 1,665 and 513,864, and their ranks were between 1 and 3,182 out of 40,330 probes [21]. From our Cbf1 Q9-PBM result, the background-subtracted intensities of 72 features which include Cbf1 motif were between 15,591 and 60,228, and their ranks were between 1 and 138 out of 131,072 probes. Although almost these features still comprise the higher intensity group in both results, Q9-PBM presents less variable intensity in the case of Cbf1.

Moreover, we applied the PBM to identify the unknown *cis*-acting element of the OsNAC6 transcription factor considered to play critical roles in stress-involved responses in *O. sativa*. OsNACc6 binds not only to 'A(A/C)GTAA', but also to G-rich motifs. We performed a gel retardation assay to validate the PBM results; these results showed that OsNAC6 can bind to either sequence, but OsNAC6 seems to displace more of 'TTACGTAAG' motif over 'CCGGGGGAG' in our experimental setup. The presence of a 'A(A/C)GTAA' motif was detected in the promoter region of rice genes directly regulated by OsNAC6.

PBM has limitations itself because some transcription factors have to be modified or multimerized after translation process in order to potentially interact with DNA. The former issue of post-translational modification could be overcome by choosing appropriate host organisms to express tagged transcription factors. The latter of multimerization is more complicate issue, however PBM is still an appropriate method if tagged proteins may sustain weak affinity to DNA by themselves. Also, there has been a concern about the position effect of a tag protein which affects the specificity of a tagged protein. It might be overcome by tagging DsRed to the other side of the protein. Our method significantly facilitated the PBM in two ways: (1) the use of quadruple 9-mers may increase protein-DNA binding interactions and (2) the one-step incubation shortens the wash and hybridization steps. The PBM with our technology will improve researchers' ability to obtain a genome-wide understanding of protein-DNA interactions.

## Conclusion

In the present paper, we demonstrated that DsRed-fused recombinant TFs can bind to their *cis*-elements, and that binding affinity can be simply detected by DsRed fluorescence intensity. Moreover, the concatenated microarray is advantageous because repeated sequences were used to elucidate the interactions between the TF and DNA motifs observed via other methods. Although some limitations (e.g., probe length, unknown interference, and stability of the fusion protein) impact these experiments, this method permits convenient identification of protein-DNA binding interactions after a simple, one-step incubation with the microarray.

## Methods

### Microarray design

The microarray was manufactured by Agilent technology (Santa Clara, CA, USA). The quadruple 9-mer protein binding microarray (Q9-PBM) consisted of 232,145 quadrupled probe features was designed which includes 131,072 features from all possible 9-mers and 101,073 replicated features out of them. Each 9-mer was concatenated four times, followed by a complementary sequence to a primer (5'-CGGAGTCACCTAGTGCAG-3') and a 5-nt thymidine linker to the slide. A microarray slide has totally 243,504 spot addresses formatted with 267 column and 912 rows. Beside the quadrupled probes, 1,474 random sequences from yeast genome, 8,081 blank features and 1,804 features from manufacturer's concern origin were included.

### Protein expression and purification

All proteins used in this study were expressed with an N-terminal fusion to a polyhistidine-tag and DsRed-monomer fluorescent protein. The coding sequence of the DsRed fluorescent protein was amplified from the pDsRed monomer vector (Clontech, Mountain View, CA, U.S.A) by polymerase chain reaction (PCR) and inserted into the pET32(a) expression vector (Novagen, San Diego, CA, USA). Full-length *Cbf1 *(Genbank accession number NC_001142) and *DREB1B *(Genbank accession number NM_118681) were amplified by PCR from the *S. cerevisiae *and *A. thaliana *genomes, respectively, and full-length *OsNAC6 *(Genbank accession number NM_001051551) was amplified from the cDNA of *O. sativa*; the sequences were then transferred to the pET32-DsRed recombinant vector. All clones were sequenced to verify the absence of mutations in the DNA-binding domains.

The proteins were expressed in *Escherichia coli *strain BL21-CodonPlus (Stratagene, La Jolla, CA, USA). Overnight cultured cells were inoculated in fresh liquid LB medium, grown at 37°C to an OD_600 _of 0.6 and induced with 1 mM isopropyl β-D-1-thiogalactopyranoside (IPTG) at 25°C for 5 h. Cell pellets were obtained by centrifugation at 4°C for 5 min at 5,000 g, resuspended and washed with cold PBS buffer including a protease inhibitor cocktail (Roche, Basel, Switzerland). Cell pellets were collected by centrifugation, resuspended in 5 ml of cold PBS buffer containing a protease inhibitor cocktail and sonicated until lysis for 5 min at 45 sec intervals on ice. The supernatant soluble fractions were retained after centrifugation at 4°C for 30 min at 9,000 g.

Proteins were verified by SDS-polyacrylamide gel electrophoresis (SDS-PAGE) and enriched using TALON resins (Clontech) adapted with immobilized metal affinity chromatography (IMAC) according to the manufacturer's protocols. The purified protein fractions were collected in a volume of 500 μl and the protein concentrations were then determined.

### Synthesis of Complementary Strands on the Microarray

The complementary DNA strand was synthesized as in a previous report [12]. Reaction solution containing 40 μM dNTP (Takara, Shiga, Japan), 1.6 μM Cy5-dUTP (GE Healthcare, Giles, UK), 1 μM 5'-CTG CAC TAG GTG ACT CCG-3' primer (Bioneer, Deajon, Korea), 1X ThermoSequenase buffer and 40 U ThermoSequenase (USB, Cleveland, Ohio, USA) was prepared. A custom-designed protein binding microarray (Agilent) was combined with the reaction solution in a hybridization chamber (Agilent) according to the manufacturer's protocol. The assembled hybridization chamber was incubated at 85°C for 10 min and then at 60°C for 90 min. The microarray was washed in phosphate buffered saline (PBS)-0.01% Triton X-100 at 37°C for 1 min, PBS-0.01% Triton X-100 at 37°C for 10 min, PBS at room temperature for 3 min and dried by centrifugation at 500 g for 2 min. The double-stranded microarray was scanned to verify successful synthesis.

### Protein Binding Microarray and Data Analysis

The double-stranded microarray was blocked with PBS-2% BSA (Sigma, St. Louis, MO, USA) for 1 h and then washed with PBS-0.1% Tween-20, PBS-0.01% Triton X-100 and PBS for 1 min. A protein binding mixture containing 200 nM protein in PBS-2% BSA, 50 ng/μl salmon-testes DNA (Sigma) and 50 μM zinc acetate was prepared. The prepared protein mixture was incubated at 25°C for 1 h for stabilization and combined with the microarray at 25°C for 1 h. The microarray was washed with PBS containing 50 μM zinc acetate and 0.5% Tween-20 for 10 min, PBS-50 μM zinc acetate-0.01% Triton X-100 for 2 min and PBS-50 μM zinc acetate for 2 min. OsNAC6 binding experiments were done in triplicated, and experiments for Cbf1, DREB1B and DsRed only were performed once.

Fluorescence images were obtained with a 4000B microarray scanner (Molecular Devices, Sunnyvale, CA, USA). Each microarray was scanned three to five times at full laser power intensity and pixel resolution 5. In order to minimize the number of saturated spots, different photomultiplier tube (PMT) gain settings were applied ranging from 550 to 780 for Cy3 and from 550 to 600 for Cy5. The fluorescence was quantified and bad spots were excluded automatically using GenePix Pro version 5.1 software (Molecular Devices). The background-subtracted median intensities were obtained and typically 0.01 - 0.05% (20 - 100) of spots showed saturated Cy3 intensity. The microarray was rescanned whenever the number of saturated spots was not in this range. The microarray data was provided with additional files (Additional file 1, the description of microarray experiment complying with the MIAME standard; Additional file 2, the raw data of DsRed-monomer PBM; Additional file 3, the processed data of DsRed-monomer PBM; Additional file 4, the raw data of CBF1 PBM; Additional file 5, the processed data of CBF1 PBM; Additional file 6, the raw data of DREB1B PBM; Additional file 7, the processed data of DREB1B PBM; Additional file 8, the first raw data of OsNAC6 PBM; Additional file 9, the first processed data of OsNAC6 PBM; Additional file 10, the second raw data of OsNAC6 PBM; Additional file 11, the second processed data of OsNAC6 PBM; Additional file 12, the third raw data of OsNAC6 PBM; Additional file 13, the third processed data of OsNAC6 PBM)

### Motif Extraction

The 29,999 single and the 101,073 replicated features of which intensity differences of two are less than 40,000 were subjected to the motif extraction. The rank-ordered signal distribution demonstrated a deep leftward slope followed by a heavy right tail (Figure 3A), as observed by Berger *et al *[12]. As the probes in the deep slope region differed by only one base, we assumed that the signal distribution was due to a specific interaction between the protein and features on the microarray. Two independent linear models, y = ax+b, were applied in the deep and the heavy right tail region using the R statistical language. In one of the examples with OsNAC6, the slope and y-axis intercept of the deep slope are -68.3 and 53074.8, respectively; those of the heavy tail are 0.0207 and 2,283, respectively. These values provide an extrapolated rank of 745 for OsNAC6. The preferred elements were extracted using the following two steps. First, feature sequences from ranks 1 to 745 were grouped using Perl script language. Any sequence that possessed at least five bases was matched; among these, at least three bases were contiguously matched to the highest one. These groups were ranked according to the highest sequence. Second, the frequency of 6-10mer was counted for each group. Frequency differences were compared and "TTACGTAA" was enriched as the highest among them. Also, our results were equivalent to the sequence defined in previous reports (e.g., 'CACGTG' for Cbf1 [16]). Additionally, the CBF1/DREB1B transcription factor bound to 'CCGAC', a C-repeat/cold- and dehydration-responsive DNA regulatory element (CRT/DRE) in Arabidopsis [17].

### Electrophoretic Mobility Shift Assay (EMSA)

Biotin-end-labeled and unlabeled oligonucleotides (Bioneer) were annealed to each complimentary sequence (see Table 1). A total of 5 μg of OsNAC6 protein was incubated with 40 fmol of biotin-labeled double-stranded oligonucleotides, 1 μg of poly dI-dC, 1× binding buffer, 2.5% glycerol and 0.05% NP40 in a 20 μl reaction volume for 30 min at room temperature according to the manufacturer's instructions (Pierce, Rockford, IL, USA). The reaction mixture was then analyzed by electrophoresis in a non-denaturing 6% polyacrylamide gel with 0.5× TBE buffer. The DNA-protein complexes in the gel were then transferred to a positively charged nylon membrane by electrophoretic transfer in 0.5× TBE at 380 mA for 30 min and cross-linked at 120 mJ/cm^2 ^using a UV-light cross-linker. Biotin was detected using the Lightshift™ Chemiluminescent EMSA kit (Pierce).

**Table 1:** Oligonucleotide sequences used for EMSA and competition studies

**Oligonucleotide**	**5'-Sequence-3'**
TTACGTAAG_forward	ACGTAAGTTACGTAAGTTACGTAAGTTACGTAAGTT
TTACGTAA_reverse	AACTTACGTAACTTACGTAACTTACGTAACTTACGT
G-rich_ forward	CCGGGGGAGCCGGGGGAGCCGGGGGAGCCGGGGGAG
G-rich_ reverse	CTCCCCCGGCTCCCCCGGCTCCCCCGGCTCCCCCGG

## Authors' contributions

T-HL designed the Q9-PBM. Y-MP constructed the OsNAC6 clone. YDC, Y-HK and H-MP provided substantial advice. M-JK performed all other experiments, which were developed and designed with the assistance of BHN and Y-KK.

## Supplementary Material

Additional file 1 **The description of microarray experiment complying with the MIAME standard**. The description of microarray experiment used to determine protein binding sequence in this work. Click here for file

Additional file 2 **The raw data of DsRed-monomer PBM**. The raw microarray data of DsRed-monomer used to verify non-binding characteristic on DNA in this work. Click here for file

Additional file 3 **The processed data of DsRed-monomer PBM**. The processed microarray data of DsRed-monomer used to verify non-binding characteristic on DNA in this work. Click here for file

Additional file 4 **The raw data of CBF1 PBM**. The raw microarray data of CBF1 used to determine binding sequence in this work. Click here for file

Additional file 5 **The processed data of CBF1 PBM**. The processed microarray data of CBF1 used to determine binding sequence in this work. Click here for file

Additional file 6 **The raw data of DREB1B PBM**. The raw microarray data of DREB1B used to determine binding sequence in this work. Click here for file

Additional file 7 **The processed data of DREB1B PBM**. The processed microarray data of DREB1B used to determine binding sequence in this work. Click here for file

Additional file 8 **The first raw data of OsNAC6 PBM**. The raw microarray data of OsNAC6 used to determine binding sequence in this work. Click here for file

Additional file 9 **The first processed data of OsNAC6 PBM**. The processed microarray data of OsNAC6 used to determine binding sequence in this work. Click here for file

Additional file 10 **The second raw data of OsNAC6 PBM**. The raw microarray data of OsNAC6 used to determine binding sequence in this work. Click here for file

Additional file 11 **The second processed data of OsNAC6 PBM**. The processed microarray data of OsNAC6 used to determine binding sequence in this work. Click here for file

Additional file 12 **The third raw data of OsNAC6 PBM**. The raw microarray data of OsNAC6 used to determine binding sequence in this work. Click here for file

Additional file 13 **The third processed data of OsNAC6 PBM**. The processed microarray data of OsNAC6 used to determine binding sequence in this work. Click here for file
